# Assessment of Hg pollution in stream waters and human health risk in areas impacted by mining activities in the Ecuadorian Amazon

**DOI:** 10.1007/s10653-023-01597-6

**Published:** 2023-05-09

**Authors:** Carlos Mestanza-Ramón, Samantha Jiménez-Oyola, Alex Vinicio Gavilanes Montoya, Danny Daniel Castillo Vizuete, Giovanni D’Orio, Juan Cedeño-Laje, Salvatore Straface

**Affiliations:** 1 Research Group YASUNI-SDC, Escuela Superior Politécnica de Chimborazo, Sede Orellana, 20001 El Coca, Ecuador; 2 Department of Environmental Engineering, University of Calabria, 87036 Rende, Italy; 3 Facultad de Ingeniería en Ciencias de La Tierra, Escuela Superior Politécnica del Litoral, ESPOL, ESPOL Polytechnic University, Campus Gustavo Galindo Km 30.5 Vía Perimetral, P.O. Box 09-01-5863, Guayaquil, Ecuador; 4 Faculty of Natural Resources, Escuela Superior Politécnica de Chimborazo, Panamericana Sur, Km 1 ½, EC-060155 Riobamba, Ecuador; 5 Department of Forest Engineering, Forest Management Planning and Terrestrial Measurements, Faculty of Silviculture and Forest Engineering, Transilvania University of Brasov, Şirul Beethoven 1, 500123 Brasov, Romania; 6 Department of Economics, Statistics ands Finasnce, University of Calabria, 87036 Arcavacata Di Rende, Italy

**Keywords:** Ecological, Human health, Biodiversity, Recreation, Agriculture, Feeding

## Abstract

Illegal gold mining activities have contributed to the release and mobilization of Hg and environmental degradation in many parts of the world. This study aims to determine the concentration of Hg in five provinces of the Amazon Region of Ecuador, in addition to assessing the risk to human health of exposed populations, applying deterministic and probabilistic methods. For this purpose, 147 water samples were collected in rivers and streams crossing and/or located near mining areas. As a result, 100% of the samples analyzed exceeded the maximum permissible limit (MPL) according to the water quality criteria for the preservation of aquatic life of the Ecuadorian regulations, while 7% of the samples exceeded the MPL for drinking water. On the other hand, considering the European Environmental Quality Standard (EQS) for surface water bodies, in our study, 100% of the samples exceed the maximum permissible limit (0.07 µg/L), and with respect to the Canadian water quality guidelines, 35% of the samples exceed the permissible limit (0.001 mg/l) for drinking water, and 100% of the samples exceed the limit for life in water bodies (0.0001 mg/l). The risk assessment revealed that the probability of developing adverse health effects from exposure to Hg is below the recommended limits according to the probabilistic assessment; this is in relation to the criterion of residential and recreational use of water resources. However, it was identified that the child population doubles the acceptable systemic risk level according to the results of the deterministic assessment in the residential scenario. This information can be used by decision-makers to implement strategies to reduce Hg contamination and exposure of the population in Ecuadorian Amazonian rivers.

## Introduction

Globally, artisanal and small-scale gold mining (ASGM) is an important source of income for rural sectors where alternatives for economic income are extremely limited (Verbrugge & Geenen, 2019). Generally, in remote sectors, this activity is developed in a rudimentary way, without compliance with environmental regulations or illegally, for example, using Hg in gold recovery processes (Niane et al., 2019). In the past decade, with the increase in demand for gold, illegal mining activities have gone from being only rudimentary to employing mechanized processes, generally financed by criminal groups, resulting in an increase in the number of people involved in gold mining (Ebus & Martinelli, 2022; Rettberg & Ortiz-Riomalo, 2016), in an increase in production and with it an increase in illegal Hg usage (Bengtsson & Hylander, 2017).

In Ecuador, ASGM dates back to colonial and pre-colonial times, reaching greater development and production in the 1990s, a period in which the existence of gold, silver and copper deposits was determined through geological studies sponsored by the Central Bank (Espinosa, 2021). Traditionally, mining activity has been artisanal and small scale; however, at present, there are consolidated medium-scale mining projects and large-scale mining activity in the process of extraction. (Agencia de Regulación y Control de Energía y Recursos Naturales no Renovables, 2022).

In the Amazon region, gold extraction has generally been carried out through rudimentary techniques using Hg illegally in the amalgamation processes (Bonotto et al., 2018). These processes have been detected in the southern zone, exactly in the jurisdiction of Chinapintza (province of Zamora Chinchipe) (Sánchez-Vázquez et al., 2016). Despite the fact that the use of Hg has been prohibited in the gold recovery process since 2010, it is still used for amalgamation in illegal mining, which represents a risk to the environment and the population.

In the Ecuadorian Amazon region, the environmental impacts caused by gold mining are an immediate consequence of illegal exploitation, and anti-technical and illegal mining practices such as indiscriminate use of Hg and inadequate tailings management (Ramírez Requelme et al., 2003b). Studies show alarming cases of the expansion of illegal mining in the provinces of Napo (Capparelli et al., 2020), Orellana (Mestanza-Ramón et al., 2022), Sucumbíos (Mora-Silva & Coronel-Espinoza, 2021; Orellana Navas et al., 2020) and Zamora Chinchipe (López-Blanco et al., 2015) whose effects on the environment are deforestation and water contamination.

The World Health Organization considers Hg as one of the most harmful heavy metals for humans and the environment (WHO, 2008). Hg can be released to the atmosphere by geogenic or anthropogenic sources and enter the natural environment as Hg-inorganic and by natural processes be transformed into Hg-organic, being the organic forms of methylmercury and dimethylmercury the most toxic ones (Fuentes-Gandara et al., 2018; Rodrigues et al., 2014). The Hg exposure pathways of greatest concern to humans will depend on the dominant species in the environment being evaluated (RAIS, 2022). The results obtained by Jiménez-Oyola et al., (2021), suggest that the greatest risk of exposure for both adults and children to inorganic Hg present in contaminated water bodies is through incidental ingestion of water. On the other hand, in work environments, where Hg amalgamation is performed, the main exposure route for people is the inhalation of elemental Hg vapors (De Miguel et al., 2014; Zhao et al., 2022). Moreover, fish consumption has been reported as an important route of entry of methylmercury into the human body, mainly in mining areas (De Miguel et al., 2014; Marrugo-Negrete et al., 2020). In this context, people living in contaminated environments have a high probability of developing adverse health effects, as they are exposed to contaminants through multiple exposure routes.

Depending on exposure, Hg can cause both acute and chronic effects; however, acute poisoning is now rare (Esdaile & Chalker, 2018). Short-term exposure to mercury Hg and its compounds can affect the respiratory tract, nervous system, kidneys and gastrointestinal tract (Li et al., 2018). Depending on the toxicity and concentration of Hg, the effects on human health may include cancer, DNA mutations, alterations in cardiac function, damage to the central nervous system and damage to blood cells and organs such as the liver, kidneys and lungs, among others (Briffa et al., 2020; Rahman et al., 2022). Among the factors that determine the toxicity of a pollutant in organisms, the following stand out: exposure route, speed of entry and excretion, distribution in the tissues and concentration in them (Briffa et al., 2020). Apart from the trophic pathway (ingestion), other forms of human exposure to heavy metals are dermal contact (Luo et al., 2022).

Human health risk assessment is a tool for estimating the risk for a population under specific exposure conditions to one or more pollutants (USEPA, 2001). Risk assessment can be performed using deterministic and probabilistic methods. The deterministic method uses a single value to represent the input variables, resulting in an estimate of risk for a study population under specific exposure conditions to one or more contaminants (Peng et al., 2016), which results in an estimated point of the risk. In contrast, the probabilistic assessment combines the probability distribution of the input parameters, resulting in a probability distribution for the resulting risk (USEPA, 2001). This procedure makes it possible to understand and manage risks and limit the exposure of vulnerable populations. This methodology has been widely used in the fields of chemical and food safety to predict or describe the effects of human exposure to toxic substances in the environment (Martín-Olmedo et al., 2016). Assessing the potential risks from the presence of contaminants in the environment is an increasingly important consideration for both decision-makers and the general public.

In a world in which human development is associated with the increase in goods and services, and the main currency of exchange for these is money (coins and banknotes), whose backing has historically been based on gold reserves, its production is increasing every day. This production brings with it socio-environmental impacts, and these are aggravated when practices are carried out illegally, informally and in an environmentally unfriendly manner. These impacts could be prevented by developing studies that allow for a timely analysis of the risks associated with extractive activities. In this sense, the objective of this research was to determine the concentration of Hg in surface water in gold mining areas in the Amazon Region of Ecuador and to evaluate the risk to human health by applying deterministic and probabilistic methods to estimate the spatial distribution pattern of socio-ecological affectation. This information can contribute to the generation of knowledge for the implementation of strategies to minimize environmental pollution in vulnerable environments.

## Materials and methods

### Study area

**Fig. 1:**
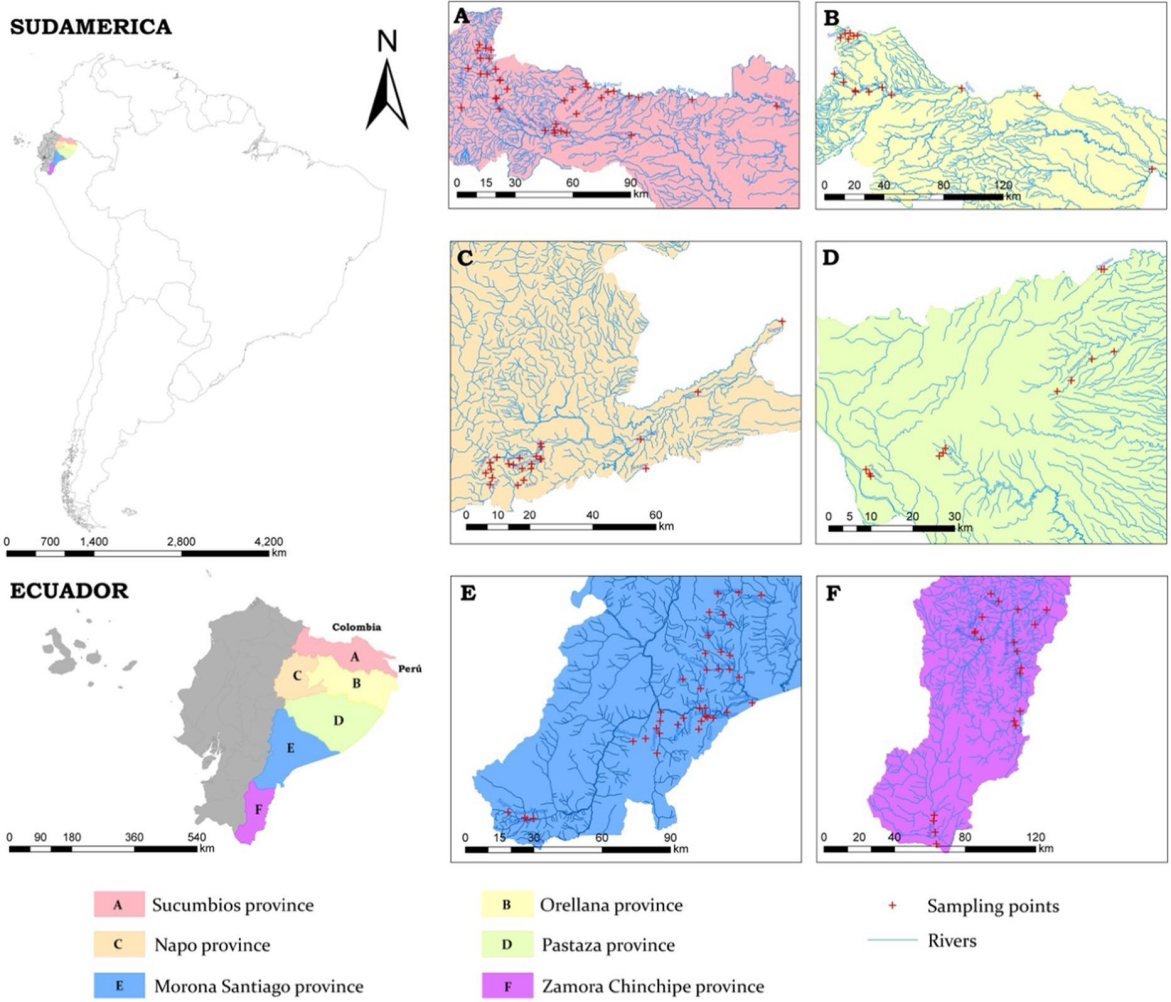
Study area and location of the sampling sites

The study was conducted in the Amazon region of Ecuador in the provinces of Sucumbíos, Orellana, Napo, Pastaza, Morona Santiago and Zamora Chinchipe (Fig. 1), with a population of 739,814 inhabitants (INEC, 2010). This area is composed of six river basins: Putumayo, Napo, Pastaza, Tigre, Morona, Santiago and Zamora Chinchipe. The main economic activities in the region are agricultural activities, extraction of natural oil resources (since 1967) and mining, which have intensified in the past two decades. These activities have been considered the main sources of environmental impacts as a result of the use of agrochemicals and the release of heavy metals and other pollutants as a result of mining and oil extraction and production processes (Capparelli et al., 2021; Maurice et al., 2019). In the study area, there are 225 mining concessions, of which two correspond to the general regime, 54 to large-scale mining (50 in the exploration stage), five to medium-scale mining and 164 to small-scale mining (Agencia de Regulación y Control de Energía y Recursos Naturales no Renovables, 2022). In addition, the area has reported a significant presence of illegal gold mining that has caused socio-environmental conflicts and has significantly affected water bodies, sediments, soil and atmosphere due to the indiscriminate discharge of mining waste and the use of Hg in the amalgamation process (López-Blanco et al., 2015; Ramírez Requelme et al., 2003).

### Sampling and laboratory analysis

Fieldwork was conducted between April and June 2022, months in which precipitation varies between 200 and 350 mm (Ilbay-Yupa et al., 2021). A total of 147 water samples were collected from rivers and streams that flow through near mining concessions (Fig. 1). The analysis focused on detecting the concentration of Hg in surface water samples from rivers and streams. Samples were collected in a 250-mL amber bottle and acidified with 0.10 mL of nitric acid. In compliance with quality policies, confidentiality and code of ethics, the samples were transported using a chain of custody to the Science Laboratory of the Escuela Superior Politécnica de Chimborazo, Sede Orellana, Ecuador. Hg content was measured by atomic absorption and hydride generation (Atomic Absorption Spectrophotometry). The Hg measurement range was from 0.0005 to 10 mg/L, the reference method used was Standard Methods, Ed. 23. 2017, 3112B—Acid digestion: EPA method 3015, 2007. Prior to analysis, samples were prepared in accordance with the nitric acid digestion procedure described in EPA Method 7473 (Mensh, 2004).

### Exposure assessment and risk characterization

The human health risk was assessed for two possible exposure scenarios: (a) residential, where the receptors adults and children are exposed through ingestion and dermal contact with water; and (b) recreational, where the receptors are exposed through incidental ingestion and dermal contact with water during recreational activities in local rivers. The average daily dose (ADD: mg/kg-day) for ingestion and dermal contact routes were calculated according to Eqs. (1) and (2), respectively (USEPA, 2001, 2004). The potential human health risk was quantified in terms of hazard quotients (HQ), for noncarcinogenic effects, by the ratio of the ADD to the reference dose (RfD_Hg_: mg/kg-day). The RfD of Hg-inorganic was chosen for the intake and dermal contact routes with water because it is the main compound soluble in water (Alfonso et al., 2010). The RfD was obtained from the Risk Assessment Information System website (RAIS, 2022).

Hazard Index (HI), which represents the cumulative noncarcinogenic risk, was estimated by the sum of the HQ_ingestion_ and HQ_dermal contact_. If HQ and HI are above 1, the safe exposure threshold is exceeded, and the systemic effects linked with the exposure can be produced.

1 $$ {\text{ADD}}_{{{\text{ingestion}} = { }}} \frac{{C_{{{\text{sw}}}} \times {\text{EF}} \times {\text{IR}} \times {\text{ED}} \times {\text{CF}}}}{{{\text{AT }} \times {\text{BW}}}} $$

2 $$ {\text{ADD}}_{{{\text{dermal contact}} = }} \frac{{C_{{{\text{sw}}}} \times {\text{EF}} \times {\text{ET}} \times {\text{ED}} \times {\text{SA}} \times {\text{kp }} \times {\text{CF}}}}{{{\text{AT}} \times {\text{BW}}}} $$

where Csw is the Hg concentration in water (mg/L); EF is the annual exposure frequency (days/year); IR is the ingestion rate of water (L/day); ET is the exposure time (hours/event); ED is the lifetime exposure duration (years); SA is the skin surface area exposed (cm2); kp is the skin permeability constant (cm/hour); AT is the averaging time (days); BW is the body weight (kg) and CF is a conversion factor.

The health risk assessment was performed by applying both the traditional deterministic method and the probabilistic method. The deterministic method performs a point estimate by employing a single value to represent the variables used in the risk equation (Peng et al., 2016). Therefore, the result is a point estimate of risk, which can be quantified based on a central tendency exposure (CTE) or a reasonable maximum exposure (RME). In contrast, probabilistic risk assessment (PRA) combines the probability distribution of one or more input parameters in the risk equation, which results in a range of values for the output risk (USEPA, 2001). The parameter values used in this study are presented in Table 1. The value of the Hg concentration at each site was used to produce the point risk maps. For concentrations < LoD, the LoD/2 was used. The risk calculation was carried out using the R free software (R Core Team, 2013), and the risk maps were made using the Geographic Information System (ArcMap 10.8.2 software).

**Table 1:** Parameters used in the risk assessment

Symbol	Units	Point estimate	Distribution	Reference
EF_residential_	day/year	350	Triangular: 345 (180–365)	Jiménez-Oyola et al. (2021)
EF_recreational_	day/year		Triangular: 120 (26–260)	Jiménez-Oyola et al. (2021)
ET_recreational_	hour/event	2.6	Triangular: 2.6 (0.5–6)	Spence & Walden (2001)
ET_residential_	hour/event	0.22	–	Jiménez-Oyola et al. (2021)
IR_residential_	L/day	*a* = 2.04, *c *= 1.28	–	Jiménez-Oyola et al. (2021)
IR_recreational_	L/event	*a* = 0.053, *c* = 0.090	–	USEPA (2011)
E	year	30	Lognormal: 11.36 ± 13.72	Israeli & Nelson (1992); Spence & Walden (2001)
ED_c_	year		Uniform: 1–6	
SA_a_	cm^2^	23,000	Normal: 18,400 ± 2300	Carr (1994); Anderson et al. (1985); Spence & Walden (2001)
SA_c_	cm^2^	7280	Normal: 6800 ± 600	
Bw_a_	kg		Normal: 72 ± 15.9	AIHC (1994); Anderson et al. (1985)
Bw_c_	kg	15.6	Normal: 15.6 ± 3.7	
*K*_*p*_	cm/hour	0.001	–	USDoE (2020)
AT	day	365 × ED	–	USEPA (2004)
RfD_oral_	mg/kg-day	0.0003	–	USDoE (2020)
RfD_dermal_	mg/kg-day	0.000021	–	

*a* = adult and *c* = children

## Results

### Hg content

**Fig. 2:**
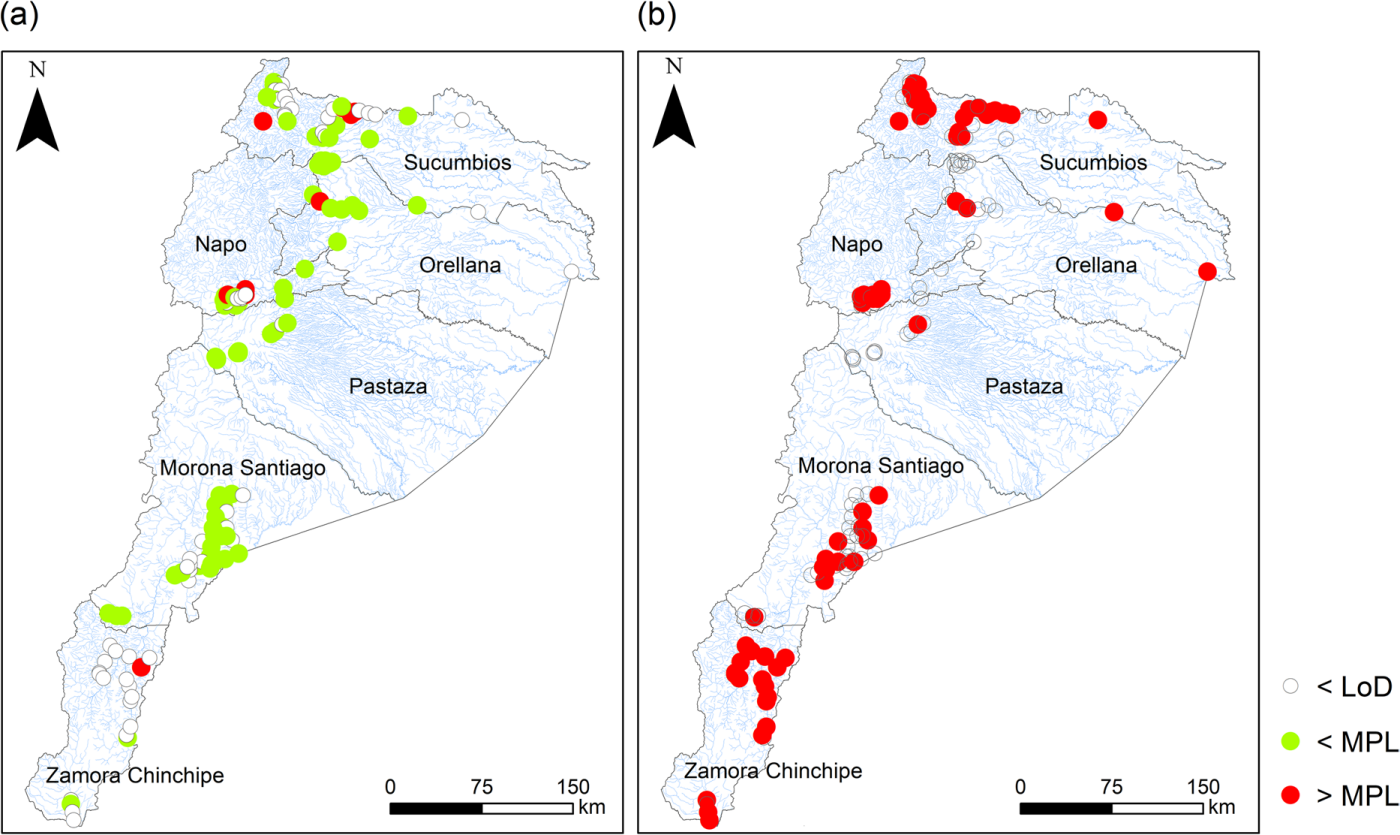
Hg concentration in water samples. The results were compared with the maximum permitted limit (MPL) according to the Water Ecuadorian Quality Guidelines for **a** drinking water and **b** for the preservation of aquatic life

The analytical results of Hg concentration in the water samples are summarized in Table 2. Fifty percent of the samples reported values below the detection limit of the measurement equipment (LoD < 0.0005 mg/L). The spatial distribution patterns of Hg concentration in comparison with the quality guidelines established by Ecuadorian legislation for drinking water and according to the admissible quality criteria for the preservation of aquatic and wild life in freshwater (INEN, 2020; MAE-TULSMA, 2015) are shown in Fig. 2. Of the samples quantified, 100% exceeded the maximum permissible limit (MPL) for water quality for the preservation of aquatic life, while 7% of the samples exceeded the MPL for drinking water quality. The high concentration of Hg measured in the rivers of the Amazon may be related to the amalgamation process that is carried out illegally in the area, and to the intensive and prolonged discharge of mining waste into the rivers (Capparelli et al., 2021; López-Blanco et al., 2015; Mora et al., 2019). In this sense, it is essential to evaluate the concentration of methylmercury in water, local fish and other foods grown in the study area, where river water can be used in agricultural fields.

**Table 2:** Hg concentration (mg/L) in water samples from the Ecuadorian Amazon

Province	*n*	Min	p50	Mean	p95	Max	S.D
Sucumbíos	36	0.0006	0.0011	0.0023	0.0078	0.0099	0.0025
Orellana	16	0.0005	0.0005	0.0011	0.0038	0.0088	0.0020
Napo	23	0.0006	0.0007	0.0025	0.0096	0.0099	0.0032
Pastaza	13	0.0005	0.0005	0.0005	0.0007	0.0011	0.0001
Morona Santiago	39	0.0005	0.0005	0.0010	0.0029	0.0041	0.0009
Zamora Chinchipe	20	0.0006	0.0013	0.0021	0.0054	0.0065	0.0018

0.006 mg/L = limit of Hg for human health protection in drinking water (INEN, 1108) 0.0002 mg/L = admissible quality criteria for the preservation of aquatic life and wildlife in fresh, marine and estuarine waters (TULSMA, 2015)

The Hg concentration detected in this study was in the range of the values reported by Capparelli et al. (2020) in the Napo River Basin. However, the concentration (p50) of Hg measured in our study was up to six times lower than that reported in the previously cited study. Furthermore, the results of our study were compared with studies conducted in the Brazilian Amazon, where gold mining is one of the main anthropogenic activities; Lino et al. (2019) reported Hg values between 0.0006 mg/L and − 0.023 mg/L in Tapajós River Basin; Vieira et al. (2018) reported Hg mean values of 0.0024 mg/L from the Rio Madeira Basin; Viana et al. (2021) reported average Hg concentrations of 0.008 mg/L and da Silva Costa et al. (2022) reported Hg values between 0.005 and 0.0085 mg/L in the Araguari River. While in the work developed by Meili et al. (1991) on Hg concentrations in Poznań surface waters, values of 20 ± 8 ng/L (range 8–40) were denoted showing statistically significant differences between the mean Hg concentrations in the river water under study. Meanwhile, Enamorado et al. (2022) highlighted that the average presence of Hg was 83 μg/L, which is above the value allowed by the World Health Organization (WHO). An important consideration is that the variation in the concentration of Hg in surface water depends on the time and magnitude of the flow, with higher contents in drier months and with lower flows (Loza del Carpio & Ccancapa Salcedo, 2020).

### 3.2. Human health risk assessment

**Fig. 3:**
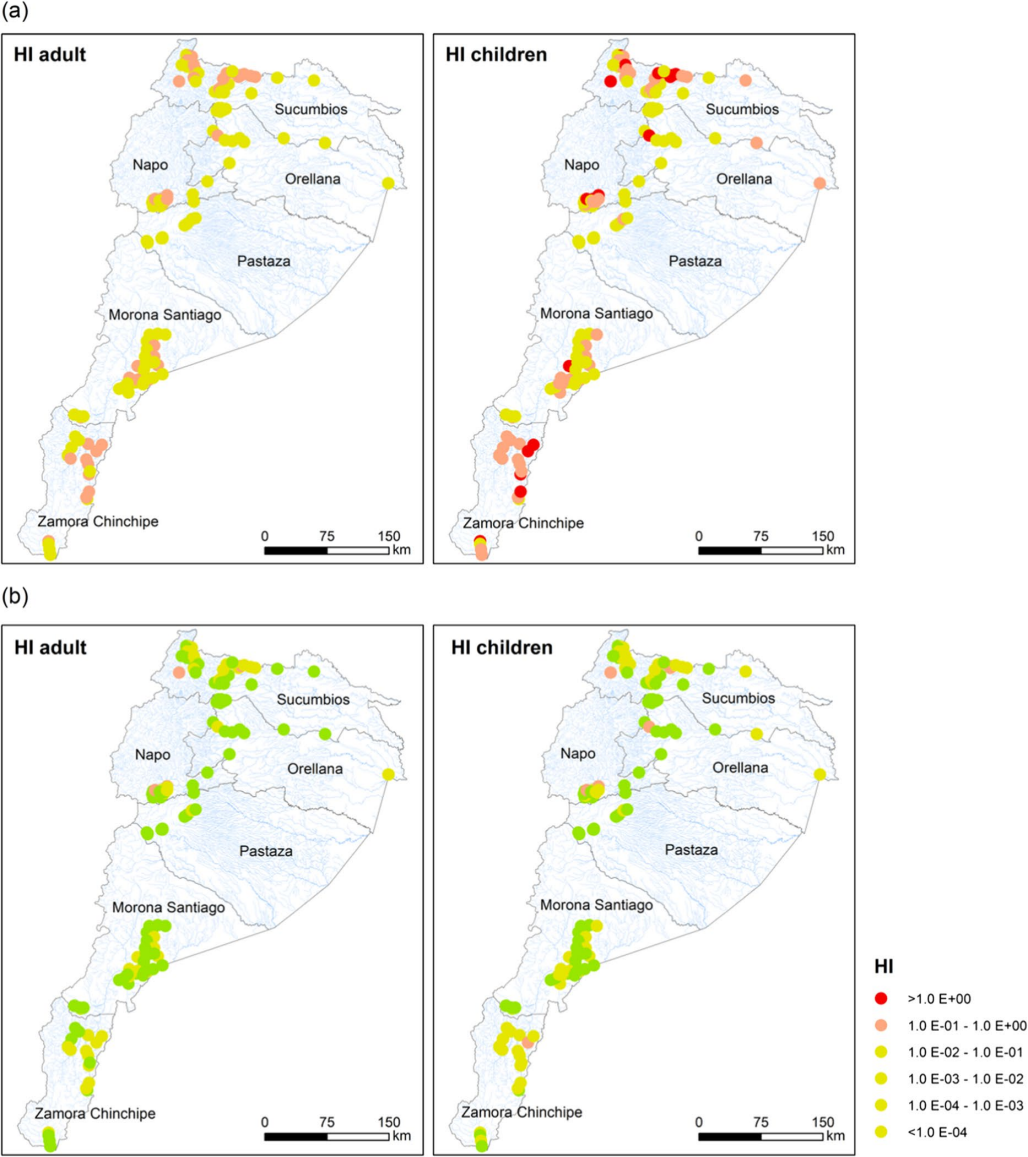
Point risk map of Hazard Index (HI) for receptors exposed to polluted surface waters in the Ecuadorian Amazon for both **a** residential and **b** recreational scenarios

The presence of Hg can cause serious health implications for residents who are exposed to this contaminant (Harada et al., 2001; Kobal et al., 2017). For this reason, the risk to human health due to Hg exposure was assessed for both adults and children living in the Amazon using deterministic and probabilistic methods. Table 3 summarizes the deterministic HI values resulting from exposure to the Hg by province for both age groups. In addition, the results of the deterministic assessment are presented in Fig. 3 as point risk maps.

**Table 3:** Deterministic HI (p95) from exposure to Hg in waters for adults and children receptors by province

	Residential scenario		Recreative scenario	
	Adults	Children	Adults	Children
Sucumbios	8.80E-01	**2.51E + 00**	1.10E-01	1.50E-01
Orellana	8.20E-01	**2.34E + 00**	9.60E-02	1.40E-01
Napo	9.00E-01	**2.56E + 00**	1.00E-01	1.50E-01
Pastaza	1.03E-01	2.90E-01	1.20E-02	1.80E-02
Morona Santiago	3.10E-01	8.80E-01	3.60E-02	5.40E-02
Zamora Chinchipe	5.50E-01	**1.58E + 00**	6.50E-02	9.80E-02

Values in bold exceed the safe exposure threshold

The point risk maps allow us to identify the sites of greatest concern to the population with respect to systemic risk scores for both residential and recreational scenarios. As shown in Fig. 3, 100% of the sites are below the threshold of acceptable risk for both receptors in the recreational scenario. In contrast, there are 20 locations where the risk assessed for the residential scenario exceeds the safe exposure threshold (HI = 1) for children, who are the most vulnerable receptors, mainly due to water ingestion. Water ingestion has been identified by several authors as one of the main entry routes of contaminants into the human body in contaminated areas (Emmanuel et al., 2022; Navoni et al., 2014). On the other hand, the dermal contact route presented HQ values in the range of 1.0E-5–1.0E-2 for both receptors, being its contribution insignificant in the evaluation of HI.

In our study, HI values that exceeded the safe exposure threshold for children were reported (in decreasing order) in the provinces of Napo > Sucumbíos > Orellana > Zamora Chinchipe. The HI results in our study were approximately half of the values presented by Jiménez-Oyola et al. (2021) in Napo Province, who reported HI = 1.85 E + 00 for adults and HI = 4.84 E + 00 for children, with surface water ingestion being the main pathway contributing to systemic risk. The main threats to human health caused by heavy metals such as Hg and potentially toxic substances have been widely documented (Osorio & Sanabria, 2020; Ralph et al., 2018; Nkuba et al., 2019; Teixeira et al., 2021; Marrugo et al., 2022). In these studies, the spatial distribution of pollution is highlighted, in addition to the identification of the danger to which human populations and the ecosystems involved are exposed (UNEP/WHO, 2008). The study by Feng et al. (2022) shows that Hg is a persistent and bio-accumulative metallic pollutant that represents a serious threat to the ecological system and human health, even in minimal concentrations. Yet more when the risk of suffering from the harmful health effects of Hg is mainly high where fish are the basis of food (Zhou et al., 2022). Our results add information on the assessment of human health risks in gold mining areas in the Amazon region. Therefore, this evidence could support the formulation of public policies to control and mitigate environmental pollution and the risks associated with the presence of potentially toxic elements in areas where gold mining is carried out without complying with environmental standards.

**Fig. 4:**
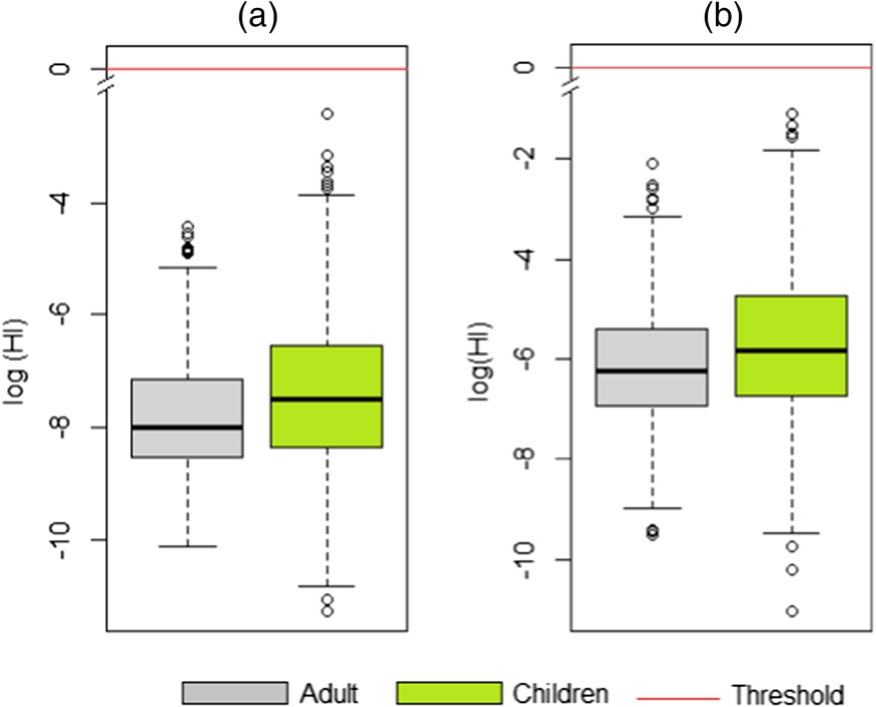
Probabilistic index (HI) related to Hg in polluted surface water for adults and children receptors for both **a** residential and **b** recreational scenarios

In order to obtain a probability distribution of risk, in this study, we performed a probabilistic risk assessment. The results estimated by the probabilistic method yielded acceptable non-cancer risk values for adult and child receptors (Fig. 4), with the maximum value of HI _adults_ = 1.21 E-02 and HI _niños_ = 8.94 E-02 in the residential scenario, and HI _adults_ = 1.23 E-01 and HI _niños_ = 3.32 E-01 in the recreational scenario. In this sense, the probability that Amazon River users may develop systemic effects from exposure to Hg through the evaluated routes is within the threshold of acceptability recommended by USEPA (2001). Similar to the results obtained by the deterministic method, the probabilistic assessment for the ingestion route presented significantly higher values; up to four orders of magnitude higher than those obtained for the dermal contact route. In addition, children reported HQ values up to three times higher than the corresponding HQ estimated in adults.

The deterministic risk outcomes were higher than the probabilistic ones. The HI_p95_ estimated by the deterministic method was 6.6 E-01 for adults and 1.9 E-01 for children for residential scenario. For recreative scenario, the HI_p95_ was 7.7 E-02 and 1.2 E-01 for adults and children, respectively. In contrast, the HI_p95_ by the probabilistic method was 3.0 E-03 for adults and 5.9 E-03 for children for residential scenario, and 1.8 E-02 for adults and 3.7 E-02 for children for recreative scenario. A study by Teixeira et al. (2021) evaluates the risk of Hg to human health revealing that the highest HQ values were mainly associated with the dermal route (HQ dermal _contact_), both for children and adults, which is due to direct contact of the contaminant with the skin surface.

Given that the results of the deterministic and probabilistic analysis differ in the risk results for the evaluated population, it is recommended that a more detailed control of the Hg content in the local rivers be carried out in order to reduce the uncertainty in the evaluation process. Likewise, it is recommended to obtain specific data on exposure of potential receptors, in order to have more conclusive results on the risk to which the Amazonian population is exposed. This is in correspondence with current national environmental regulations that promote the management of water resources through public, private and community intervention (National Assembly of Ecuador, 2014).

Despite the results obtained by the probabilistic method, it is important to consider that there are other potential routes of risk for the population, such as the ingestion of local fish, as these may have significant amounts of methylmercury in their tissues (Webb et al., 2004) or the consumption of locally grown food (irrigated with water from rivers with high Hg content). It is now known that even at relatively low concentrations, Hg in the environment can lead to elevated levels of methylmercury in food, posing a potential risk to consumers (Fuentes-Gandara et al., 2018). Additionally, exposure to Hg-contaminated sediments has also been reported as an important route of exposure for residents of the Ecuadorian Amazon (Jiménez-Oyola et al., 2021; Mora et al., 2019; Ramírez Requelme et al., 2003a). Therefore, further studies are needed to understand the mechanisms governing the mobility of Hg in Amazonian rivers contaminated by mining activity and the associated risks to adults and children. In addition to mining activity, other potential sources of anthropogenic contamination in the area are; urban pollution, fish farming, non-functional landfill areas and oil activities (Capparelli et al., 2020; Coronel Vargas, Au & Izzotti, 2020; Maurice et al., 2019). Therefore, a comprehensive risk assessment should be conducted, analyzing exposure to multiple potentially toxic elements (e.g., As, Cd and Cr) generated by the aforementioned activities.

## Discussion

The results of this study present an alarming scenario regarding the presence of Hg in the Ecuadorian Amazon. The highest concentrations of Hg were reported in the provinces of Sucumbíos, Napo and Orellana, located to the north of the study area, where concentrations exceeded up to 1.7 times what is established in the Ecuadorian regulations for drinking water quality. With respect to water quality for the preservation of aquatic life, the situation is more serious, since Hg exceeded up to 50 times what is established in the Ecuadorian regulations in the provinces analyzed. Considering these results, wildlife would be exposed to a greater impact, putting sensitive ecosystems and species at risk in the Ecuadorian Amazon. On the other hand, regarding the risk to human health, although there is no evidence of a high risk to the population of the provinces analyzed, it was determined that children are the most exposed to the risk, especially in the populations of Sucumbíos, Orellana, Napo, Morona Santiago and Zamora Chinchipe. For this reason, the exposure of the child population should be controlled in order to maintain risk levels within acceptable limits. As a result, 100% of the samples analyzed exceeded the maximum permissible limit (MPL) according to the water quality criteria for the preservation of aquatic life of the Ecuadorian regulations, while 7% of the samples exceeded the MPL for drinking water. On the other hand, considering the European Environmental Quality Standard (EQS) for surface water bodies (Jirka et al., 2004), in our study, 100% of the samples exceed the maximum permissible limit (0.07 µg/L), and with respect to the Canadian water quality guidelines (Spry & Branch, 2015), 35% of the samples exceed the permissible limit (0.001 mg/L) for drinking water, and 100% of the samples exceed the limit for life in water bodies (0.0001 mg/L).

The Hg content in the analyzed samples could be directly related to the presence of illegal gold mining activity, mainly in the northern part of the study area, whose effects on the environment are the following (Capparelli et al., 2020; Mestanza-Ramón et al., 2022; Mora-Silva & Coronel-Espinoza, 2021; Orellana Navas et al., 2020), whose effects of this activity on the environment are deforestation and water pollution. One of the main factors that have allowed the increase in illegal mining activities, intrinsically linked to environmental contamination, has been the inability of the state in the mining control and monitoring processes. Despite having legal sanctioning instruments (administrative and criminal), these have not had an effective application in the eradication of the problem of Hg usage and in the control of the environmental impact caused by illegal miners.

In the criminal sphere, in 2014, the illicit activity of mineral resources, the financing or supply of machinery for the illicit extraction of resources and environmental damage were criminalized. (Asamblea Nacional Constituyente del Ecuador, 2014). However, there has been no evidence of a significant impact of the applicability of this legal instrument in reducing illegal mining activities in Ecuador.

The lack of personnel and technical–operational capacity of those in charge of mining control, the lack of security in the face of threats from the mafias that control illegal mining activity and internal corruption in state entities are some of the factors that have contributed to this problem being maintained over the years. In Ecuador, there are areas where illegal mining activities are of public knowledge; however, there is no decision by the control entities for the implementation of definitive actions to eradicate this practice so harmful for both the population and the environment. This type of situation is recurrent and has been widely commented in developing countries. With respect to Latin America, similar cases have been reported in Venezuela (Santos-Francés et al., 2011), Brazil (Castilhos et al., 2015; Lino et al., 2019), Colombia (Cordy et al., 2011; Restrepo et al., 2021) and Peru (Cruzado-Tafur et al., 2021), where illegal mining activities and the indiscriminate use of Hg have caused severe environmental and health impacts on the populations.

In this context, it is necessary for the Ecuadorian government to focus its efforts on the processes of control and monitoring of gold mining activity, at all scales, considering that it is possible to bring together an economic benefit for the state and a sustainable use of natural resources, in an environmentally friendly and responsible way with local communities.

## Conclusions

This study evaluated the concentration of Hg in surface water in five provinces of the Amazon region of Ecuador and identified the potential risk to the inhabitants’ health of the study area. All of the samples analyzed did not meet the water quality criteria for the preservation of aquatic life according to Ecuadorian regulations. The probabilistic risk assessment revealed that the probability of developing adverse human health effects from Hg exposure is below the permissible limits for adult and child receptors in both residential and recreational settings. In contrast, it was identified that the child population doubles the acceptable risk level for the deterministic risk assessment in the residential setting. In addition, this study allowed the identification of sites that represent a potential systemic risk, mainly for the child population, due to surface water ingestion. Therefore, these findings denote the importance of incorporating a spatial distribution pattern of social and ecological affectation in the study area. Likewise, the need to deepen the investigation of anthropogenic contamination in the Ecuadorian Amazon and to evaluate the concentration and bioavailability of Hg, methylmercury and other potentially toxic elements in different matrices such as sediments, soils and food (fish or local crops), in order to carry out an integral evaluation of the risks to human health, is evident. The results of this study can support a participatory approach in the design of strategies and policies for water use and control of anthropogenic pollution. Likewise, they can constitute a baseline in risk assessment and management issues for the inhabitants of the Ecuadorian Amazon and other regions of Ecuador with similar problems of contamination by heavy metals and metalloids.
